# Plurihormonal cells of normal anterior pituitary: Facts and conclusions

**DOI:** 10.18632/oncotarget.16502

**Published:** 2017-03-23

**Authors:** Lubov B. Mitrofanova, Petr V. Konovalov, Julia S. Krylova, Victoria O. Polyakova, Igor M. Kvetnoy

**Affiliations:** ^1^ Federal Almazov North-West Medical Research Center, St. Petersburg, 197341, Russian Federation; ^2^ Ott Research Institute of Obstetrics, Gynecology and Reproductology, St. Petersburg, 199034, Russian Federation

**Keywords:** pluriihormonality of normal anterior pituitary, confocal laser scanning microscopy, pituitary adenoma

## Abstract

**Introduction:**

plurihormonality of pituitary adenomas is an ability of adenoma cells to produce more than one hormone. After the immunohistochemical analysis had become a routine part of the morphological study, a great number of adenomas appeared to be multihormonal in actual practice. We hypothesize that the same cells of a normal pituitary gland releases several hormones simultaneously.

**Objective:**

To analyse a possible co-expression of hormones by the cells of the normal anterior pituitary of adult humans in autopsy material.

**Materials and methods:**

We studied 10 pituitary glands of 4 women and 6 men with cardiovascular and oncological diseases. Double staining immunohistochemistry using 11 hormone combinations was performed in all the cases. These combinations were: prolactin/thyroid-stimulating hormone (TSH), prolactin/luteinizing hormone (LH), prolactin/follicle-stimulating hormone (FSH), prolactin/adrenocorticotropic hormone (ACTH), growth hormone (GH)/TSH, GH/LH, GH/FSH, GH/ACTH, TSH/LH, TSH/FSH, TSH/ACTH. Laser Confocal Scanning Microscopy with a mixture of primary antibodies was performed in 2 cases. These mixtures were ACTH/prolactin, FSH/prolactin, TSH/prolactin, ACTH/GH, and FSH/GH.

**Results:**

We found that the same cells of the normal adenohypophysis can co-express prolactin with ACTH, TSH, FSH, LH; GH with ACTH, TSH, FSH, LH, and TSH with ACTH, FSH, LH. The comparison of the average co-expression coefficients of prolactin, GH and TSH with other hormones showed that the TSH co-expression coefficient was significantly the least (9,5±6,9%; 9,6±7,8%; 1,0±1,3% correspondingly).

**Conclusion:**

Plurihormonality of normal adenohypophysis is an actually existing phenomenon. Identification of different hormones in pituitary adenomas enables to find new ways to improve both diagnostic process and targeted treatment.

## INTRODUCTION

Plurihormonality of pituitary adenomas is the ability of adenoma cells to produce more than one hormone. After the immunohistochemical analysis has become a routine part of the morphological study, a great number of adenomas appear to be multihormonal in real practice [1]. However, according to WHO's recommendations, the gonadotropinomas releasing luteinizing hormone (LH) and follicle-stimulating hormone (FSH), along with the prolactin secreting adenomas releasing growth hormone (GH) and thyroid-stimulating hormone (TSH) are not considered to be plurihormonal [2].

The pituitary gland develops from 2 of different origin: an upward invagination in the roof of the primitive oral cavity (Rathke's pouch) and an evagination from the floor of the third ventricle (pituitary stalk). Over several weeks the adenohypophysis develops from the anterior wall of Rathke's pouch, and the intermediate lobe of the pituitary gland develops from its posterior wall. The posterior pituitary and the stalk of the pituitary gland develop from a vertical evagination from the floor of the third ventricle. By this means the adenohypophysis is a derivative of epithelial hypophyseal pouch and is the anterior lobe of the pituitary gland. It is comprised of adenomeres consisting of secretory cells which are covered with reticular fibers and capillaries [3].

There are 5 types of cells in the adenohypophysis: somatotrophs, prolactin cells, corticotropic cells, thyrotropic cells and gonadotropic ones. Corticotrophs produce ACTH and the other hormones which are derivatives of pro-opiomelanocortin (POMC), thyrotropic cells produce TTH and gonadotrophs produce FSH and LH.

Somatotrophs comprise almost 50% of the pituitary cells of the anterior pituitary. They are located predominantly in the lateral wings of the pituitary gland. 15-20% of pituitary cells are lactotrophs, or the cells producing prolactin. They can most often be found in the posterolateral region of the pituitary gland. Thyrotrophs make up about 5% of the anterior pituitary cells. Thyrotrophs are located in the anteromedial and anterolateral aspects of the pituitary gland. Corticotrophs are concentrated in the anteromedial areas of the pituitary gland and make up about 15-20% of all the anterior pituitary cells. Gonadotrophs constitute approximately 10-15% of anterior pituitary cells. They are located everywhere within the anterior lobe of pituitary, but their predominant quantity is found in the lateral wings of the pituitary gland. Immunohistochemical examination of anterior pituitary cells showed that a part of anterior pituitary cells have no hormone immunoreactivity, though electron microscopy reveals that secretory granules present in their cytoplasm. These cells used to be called chromophobes, and according to the new classification they are named null cells. Non-functioning pituitary adenomas are considered to be composed of these cells. Some researchers think that null cells are not involved in secreting hormones but are only the source which produces other cell types of the anterior pituitary. The anterior pituitary gland secretes 6 principle hormones that can be divided into 3 groups: 1) protein hormones belonging to the family of somatomammotrophins, namely, GH and prolactin; 2) glycoproteins, such as FSH, LH and TSH; 3) hormones derived from POMC which are ACTH, lipotropins, melanostimulating hormone, endorphins, and hormones belonging to polypeptides.

It is yet to be explained whether plurihormonal cells are more often found in the normal pituitary gland or in adenomas [4]. Under physiological conditions the presence of plurihormonal cells can be related to trans-differentiation which means the conversion of one cell type into another [5].

Literature review has demonstrated that it is not clear if the cells of the normal anterior pituitary in adults co-express some hormones simultaneously and what hormones they are in the case of co-expression. We hypothesize that the same cells of a normal pituitary gland release several hormones simultaneously.

## RESULTS

**Figure 1:**
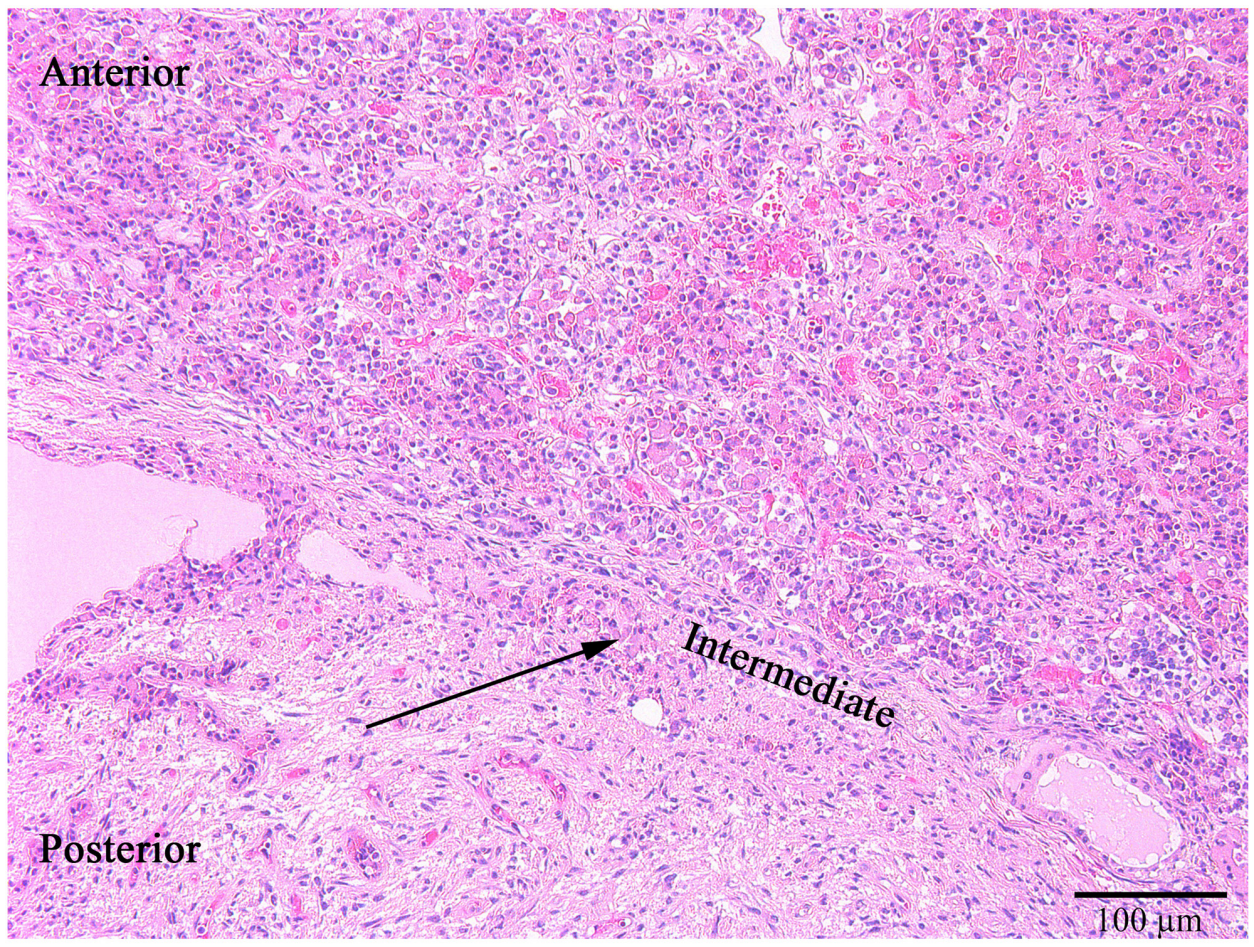
The anterior, intermediate (thin black arrow) and posterior lobes of the normal pituitary gland Hematoxylin and eosin, x100.

**Figure 2:**
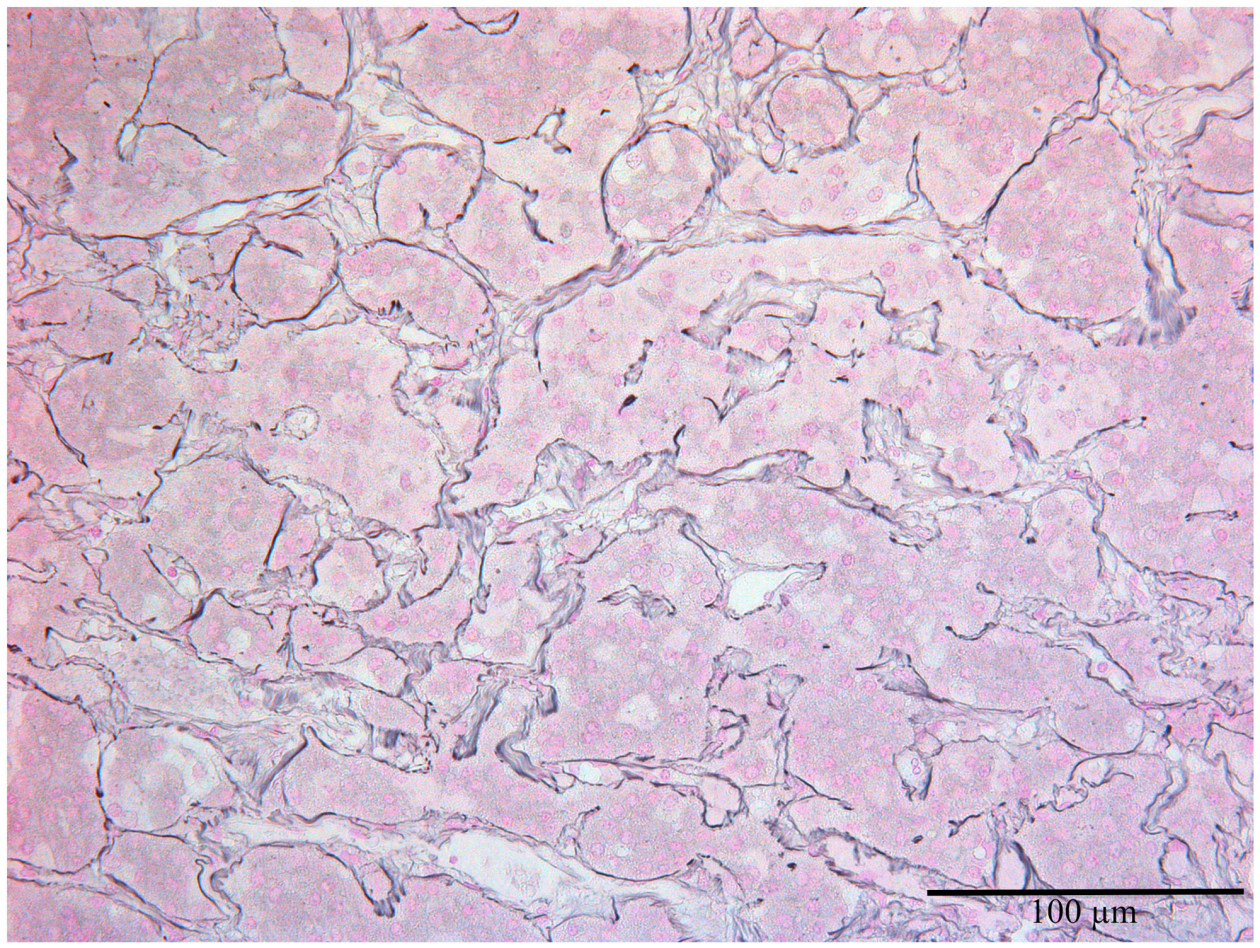
The normal anterior pituitary, the Gordon and Sweet's silver staining for reticular fibers (black), x200

The length, the width and the height of the pituitary glands were 9-15 mm, 5-10 mm and 6-8 mm correspondingly. Their anterior, intermediate and posterior lobes were clearly recognized by histological examination (Figure 1). Anterior pituitary comprised 70-75% of the total pituitary gland. It consisted of many glandular epithelial cells arranged in cords and clusters which were covered with reticular fibres and capillaries of trabeculae or adenomeres. The trabecular structure of anterior pituitary was displayed well using the Gordon and Sweet's silver staining method (Figure 2).

**Figure 3:**
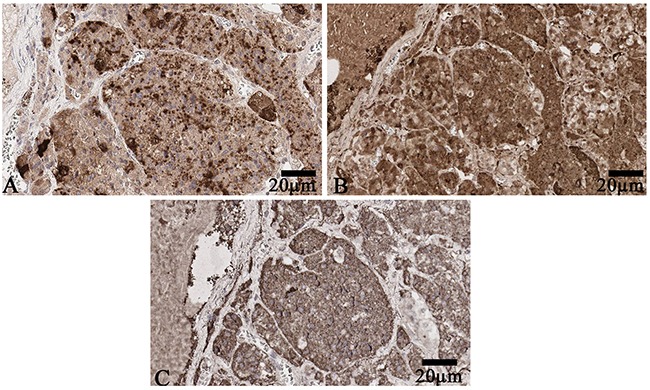
**(A) Luteinizing hormone expression in pars distalis of anterior pituitary**. Scanned specimen, x200. **(B)** Adrenocorticotropic hormone expression in pars distalis of anterior pituitary. Scanned specimen, x200. **(C)** Growth hormone expression in pars distalis of anterior pituitary. Scanned specimen, x200.

All 10 anterior pituitary glands released all 6 hormones being investigated. Expression of all these hormones was observed in cytoplasm of pituitary cells. Having compared the scanned immunochemical sections of 10 pituitary glands, we found out evident overlaps of expression areas of 4 hormones, namely GH, prolactin, LH and ACTH, in pars distalis (Figure 3).

**Figure 4:**
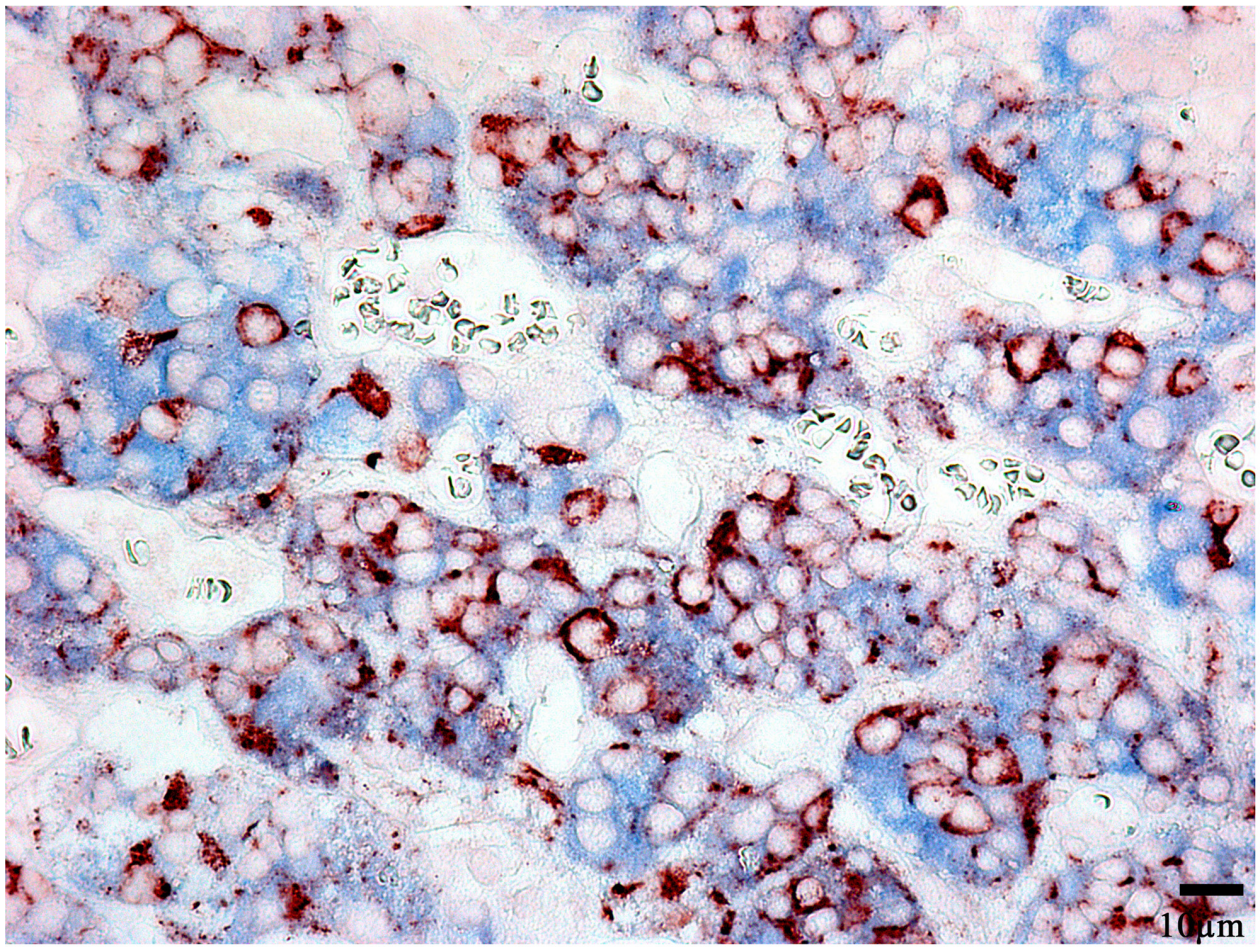
Double-staining immunohistochemistry of the normal anterior pituitary Prolactin/Thyroid-stimulating hormone; x 400. Prolactin is visualized with red colour, Thyroid-stimulating hormone with blue colour, hormone co-expression with marron.

**Figure 5:**
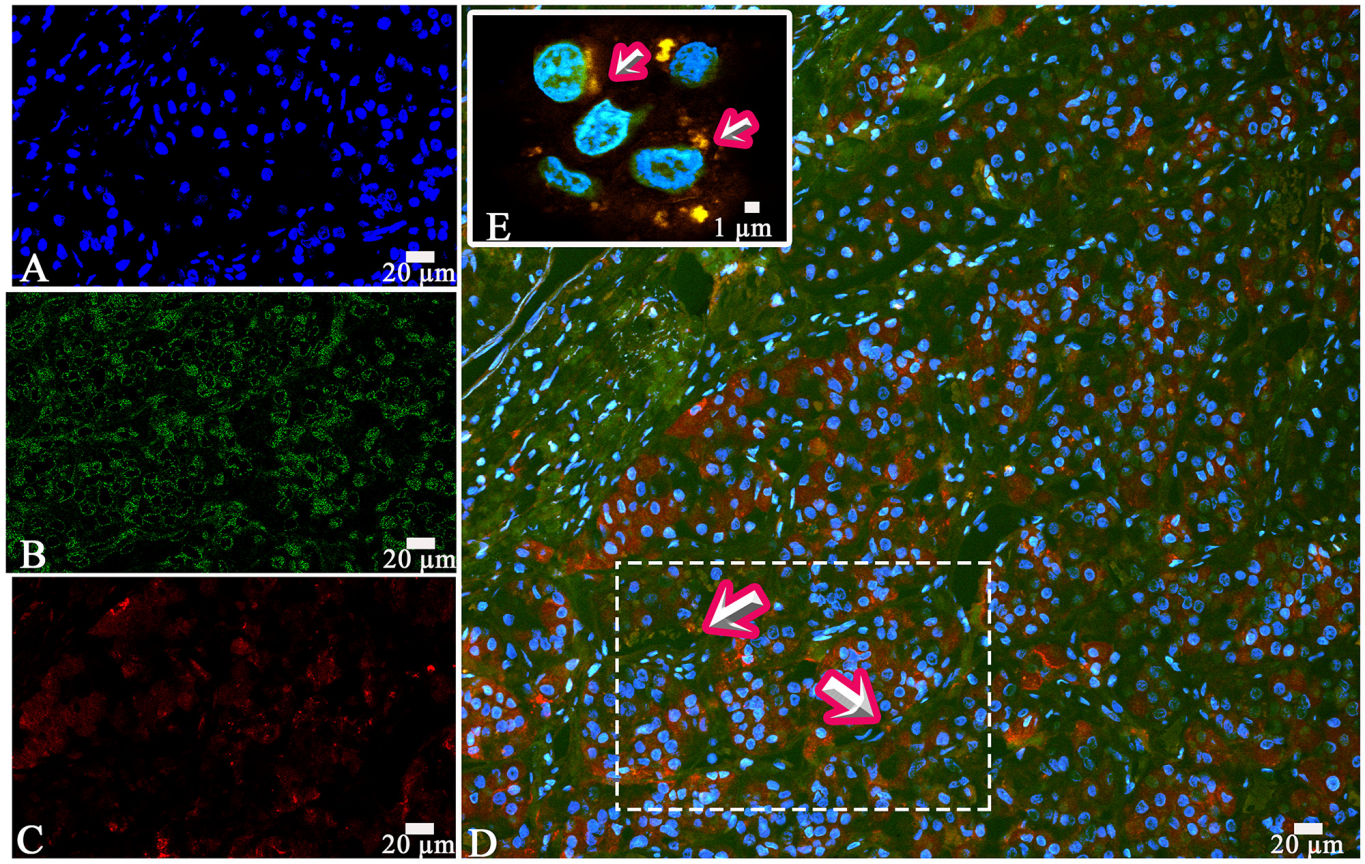
Laser Confocal Scanning Microscopy of the normal anterior pituitary **(A)** Blue fluorescence of cell nuclei (DAPI). **(B)** Green fluorescence of prolactin. **(C)** Red fluorescence of Thyroid-stimulating hormone. **(D, E)** Hormone co-expression is visualized with yellow/orange colour (arrows). A, B, C, D x200. E x600.

Prolactin co-released in the same cells with other hormones in all 10 cases. Light microscopic morphometry showed that the co-expression coefficient of prolactin and TSH varied between 3.4% and 22%. Also on light microscopic examination the prolactin and TSH co-expression was observed in 13±8 % of all the anterior pituitary cells on the average (Figure 4, Table 1). Doubled-stained cells were recognized in all the regions of anterior pituitary. Laser confocal scanning microscopy (CLSM) revealed a highly prominent co-expression of these hormones in 50% of the cells of pars distalis (Figure 5).

**Table 1:** The average percentages of anterior pituitary hormone co-expression

Hormone mixture	The average percentage of co-expression, %
GH/ACTH	7,32±6,3
GH/FSH	12,8±10,8
GH/LH	11,7±8,4
GH/TSH	6,38±5,4
PRL/TSH	13,08±8,9
PRL/LH	7,7±4,5
PRL/FSH	8,2±4,9
PRL/ACTH	9,2±9,2
TSH/FSH	1,06±0,8
TSH/ACTH	1,12±1,4
TSH/LH	0,86±1,04

PRL - prolactin, GH - growth hormone, ACTH - adrenocorticotropic hormone, LH - luteinizing hormone, FSH - follicle-stimulating hormone, TSH - thyroid-stimulating hormone

**Figure 6:**
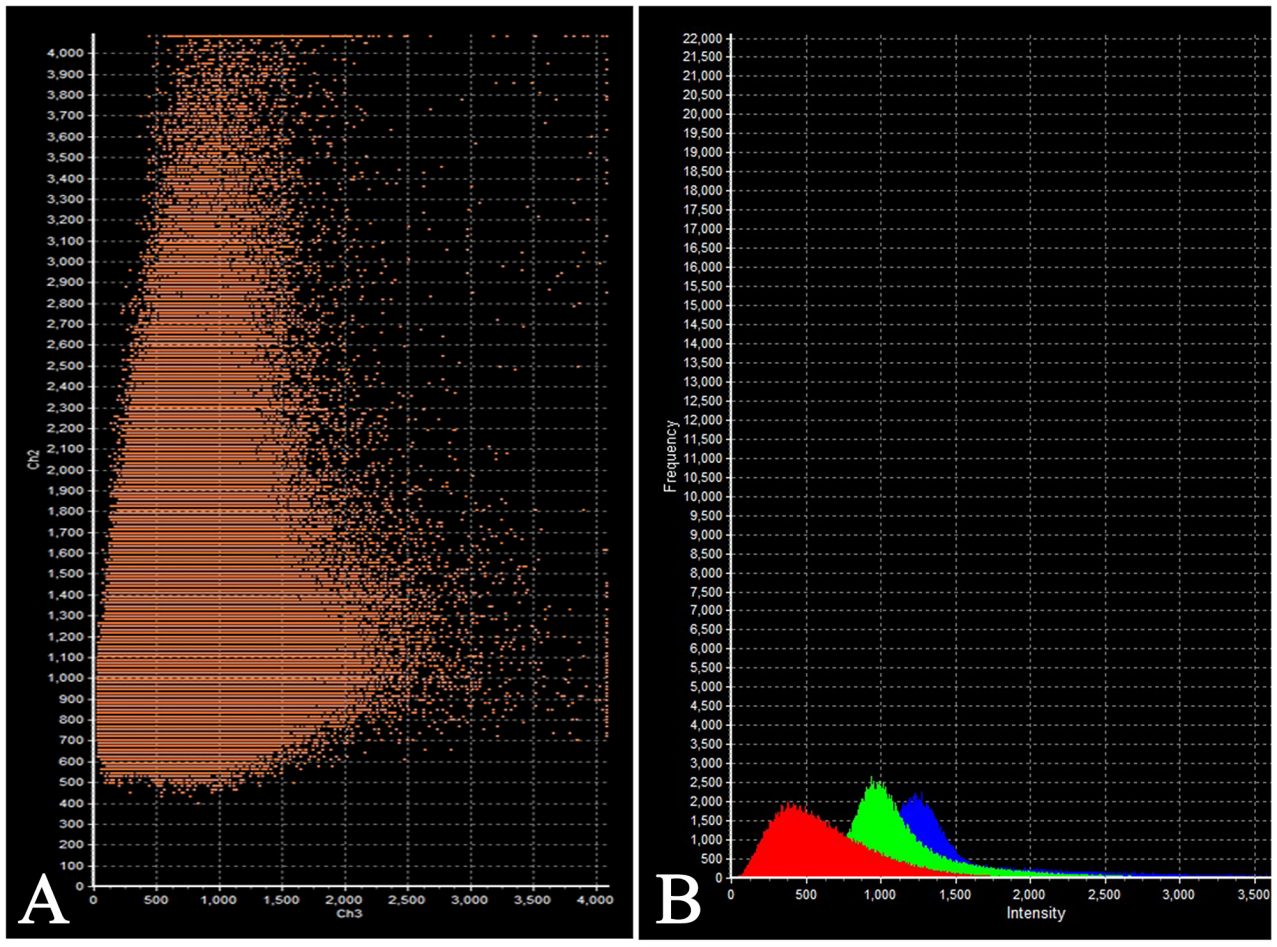
Laser Confocal Scanning Microscopy of the normal anterior pituitary **(A)** Scatterplot of red (Thyroid-stimulating hormone) and green (Prolactin) pixel intensities of pituitary cells. **(B)** Intensity Histogram of red (Thyroid-stimulating hormone), green (Prolactin) and blue (DAPI) fluorescence.

The intensity of expression (fluorescence) of TSH was 655 - 1540 standard units compared to 1204 – 1324 standard units for prolactin (Figure 6, Table 2).

**Table 2:** Intensity of fluorescence (hormone expression). Laser Confocal Scanning Microscopy

Hormone combination	Intensity (in standard units):	
	maximum	minimal
Prolactin/Thyroid-stimulating hormone:		
Prolactin	1324	1204
Thyroid-stimulating hormone	1540	655
Prolactin/Follicle-stimulating hormone:		
Prolactin	1589	885
Follicle-stimulating hormone	726	617
Prolactin/Adrenocorticotropic hormone:		
Prolactin	704	681
Adrenocorticotropic hormone	604	324
Growth hormone/Adrenocorticotropic hormone:		
Growth hormone	736	662
Adrenocorticotropic hormone	613	452
Growth hormone/Follicle-stimulating hormone:		
Growth hormone	729	565
Follicle-stimulating hormone	445	163

**Figure 7:**
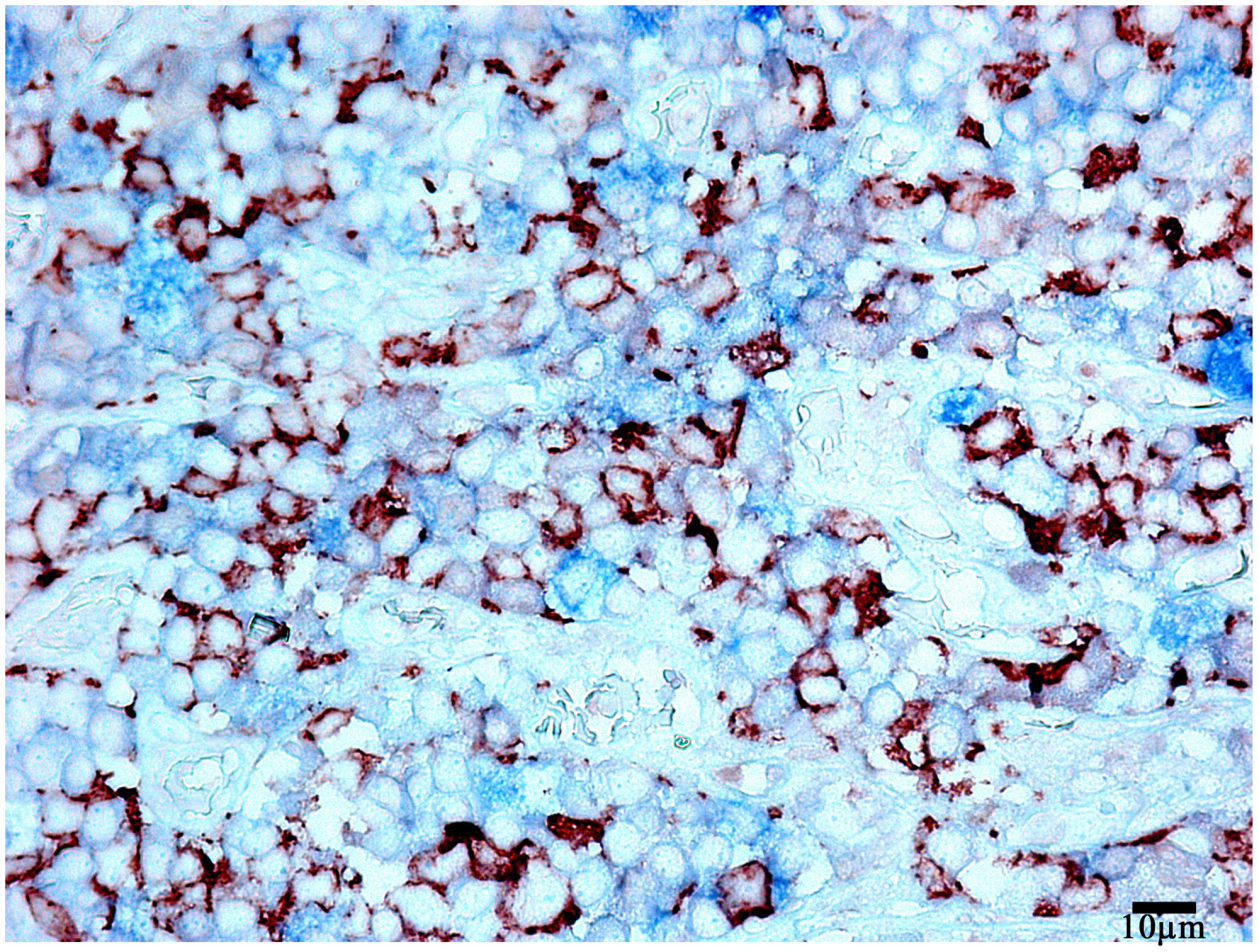
Double-staining immunohistochemistry of the normal anterior pituitary Prolactin/Luteinizing hormone, x 400. Prolactin is visualized with red colour, Luteinizing hormone with blue colour, hormone co-expression with marron.

The co-expression coefficient of prolactin and LH varied between 4 and 15 % in different patients. The diffuse co-expression of these hormones was observed all over the anterior pituitary and made up 7,7±4,5% of all the cells on the light microscopy (Figure 7). Likewise, the diffuse co-expression of prolactin and FSH was observed all over the anterior pituitary. The co-expression coefficient varied between 3 and 15% and on the average it was 8,2±4,9%.

**Figure 8:**
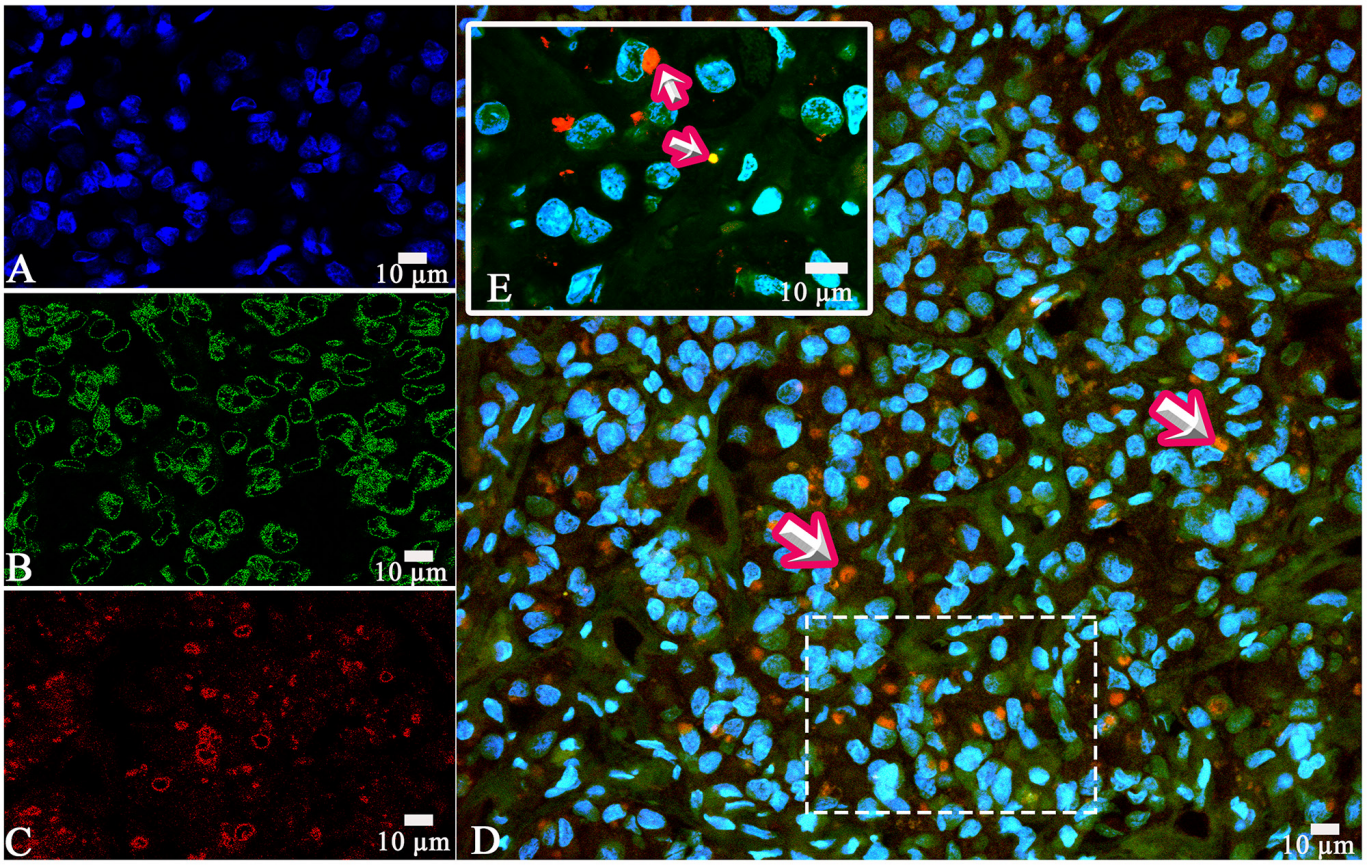
Laser Confocal Scanning Microscopy of the normal anterior pituitary **(A)** Blue fluorescence of cell nuclei (DAPI). **(B)** Green fluorescence of prolactin. **(C)**. Red fluorescence of Follicle-stimulating hormone. **(D, E)** Hormone co-expression is visualized with yellow/orange colour (arrows). A, B, C, D x400. E x600.

**Figure 9:**
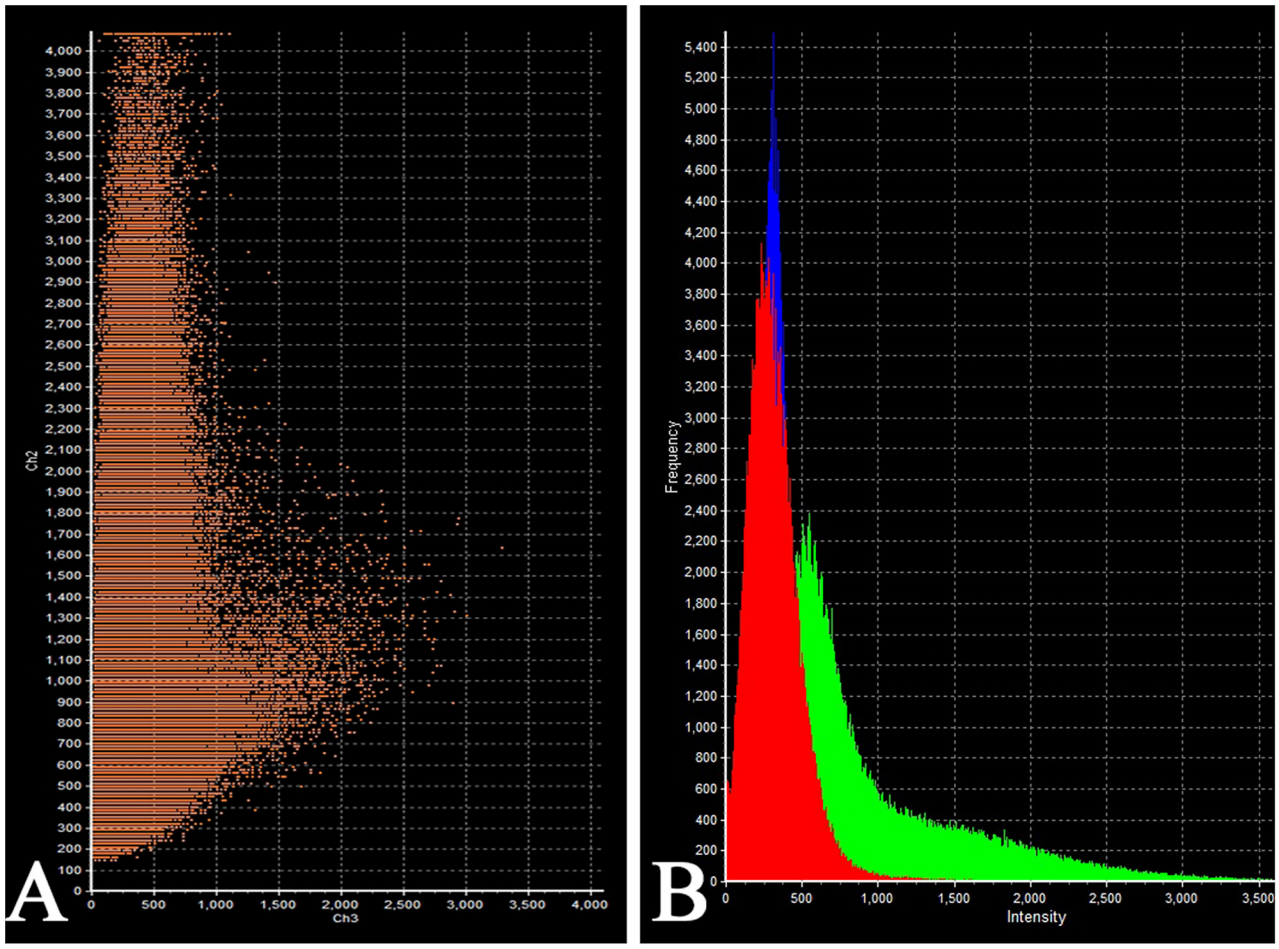
Laser Confocal Scanning Microscopy of the normal anterior pituitary **(A)** Scatterplot of red (Follicle-stimulating hormone) and green (Prolactin) pixel intensities of pituitary cells. **(B)** Intensity Histogram of red (Follicle-stimulating hormone), green (Prolactin) and blue (DAPI) fluorescence.

On the confocal microscopy the prolactin/FSH double-staining was most prominent in the pars distalis and was observed in 51% of the cells (Figure 8). The intensity of FSH expression (fluorescence) was 617 - 726 standard units compared to 885 – 1589 standard units for prolactine (Figure 9).

**Figure 10:**
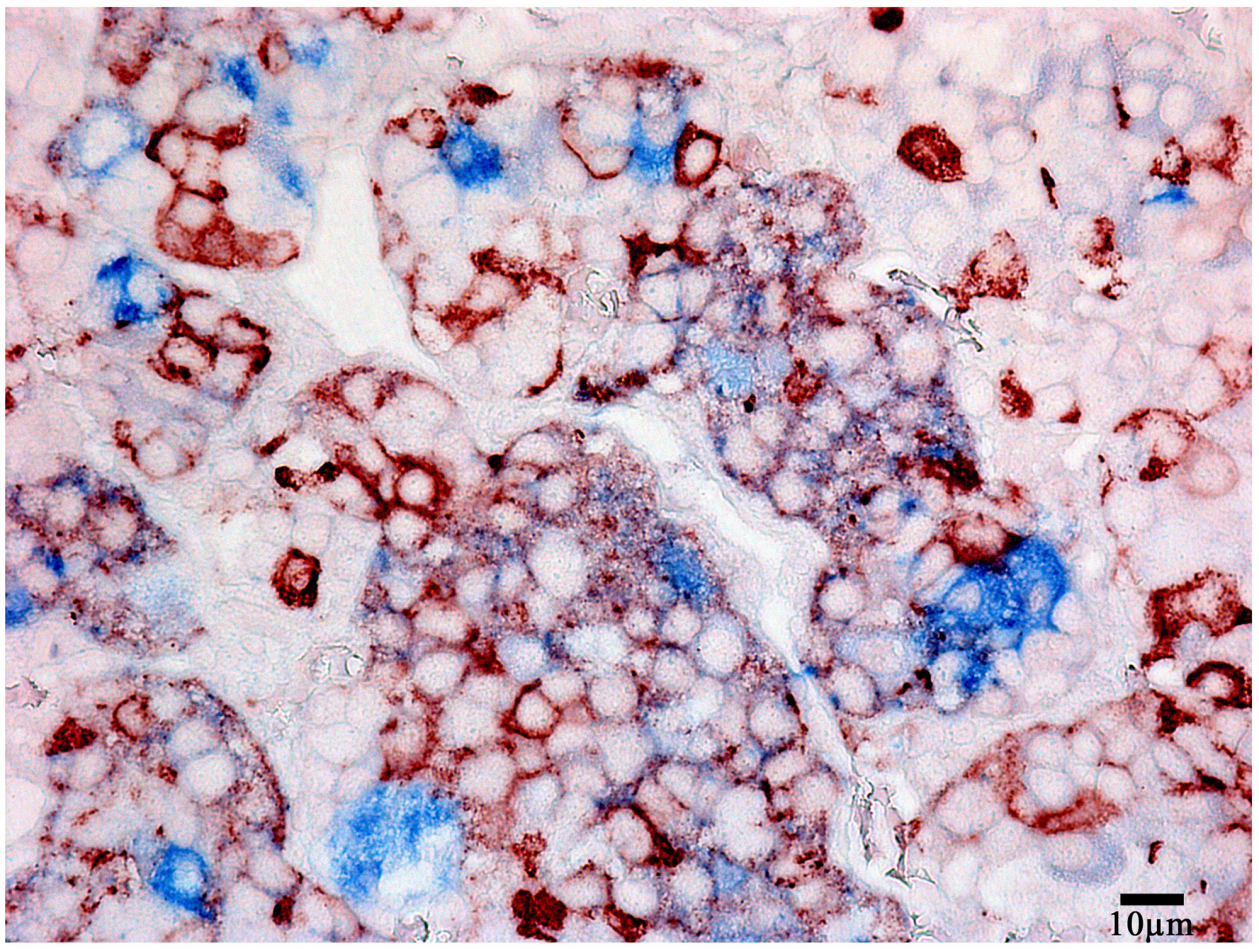
Double-staining immunohistochemistry of the normal anterior pituitary Prolactin/Adrenocorticotropic hormone), x 400. Prolactin is visualized with red colour, Adrenocorticotropic hormone with blue colour, hormone co-expression with marron.

**Figure 11:**
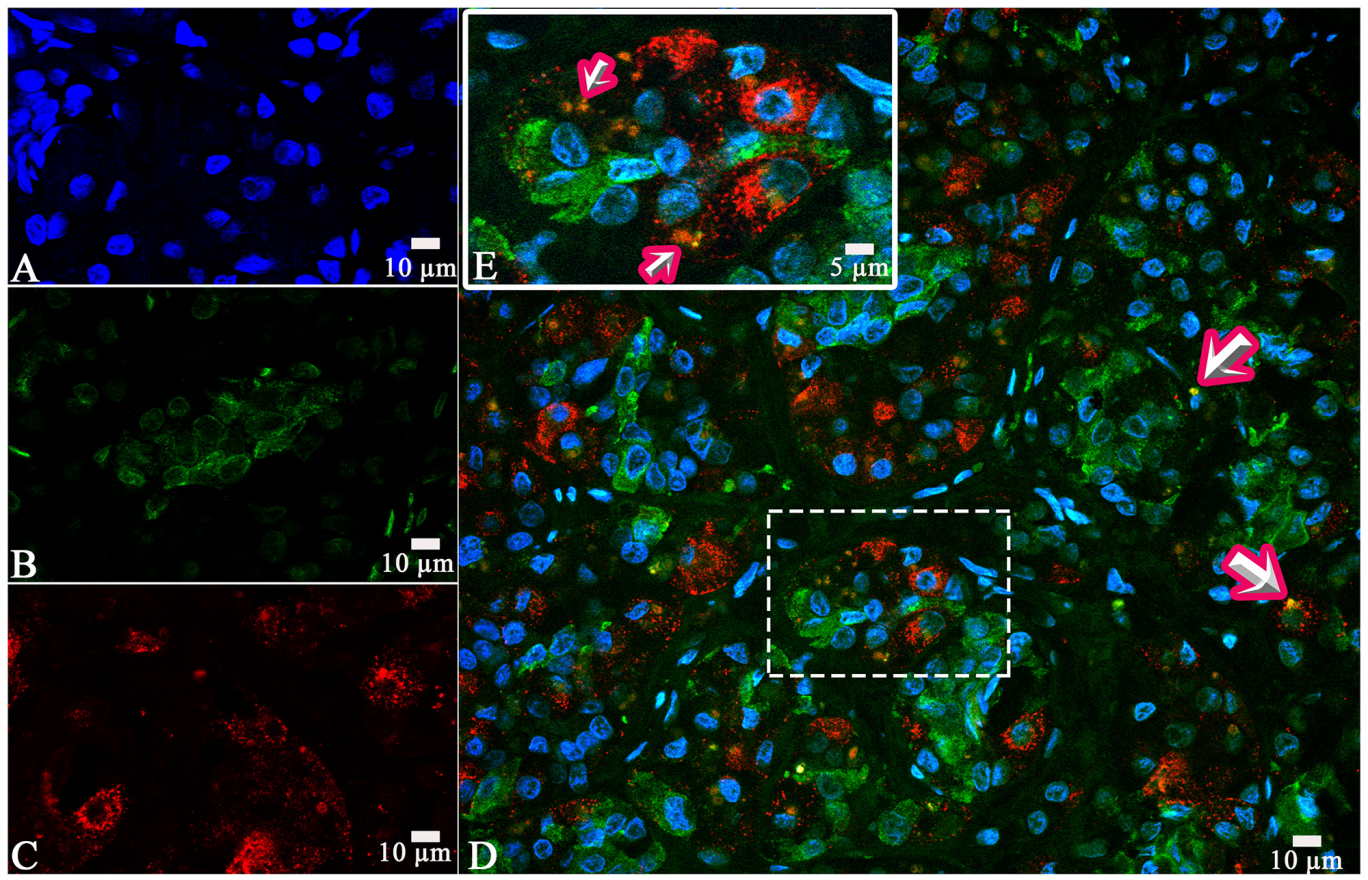
Laser Confocal Scanning Microscopy of the normal anterior pituitary **(A)** Blue fluorescence of cell nuclei (DAPI). **(B)** Green fluorescence of prolactin. **(C)** Red fluorescence of Adrenocorticotropic hormone. **(D, E)** Hormone co-expression is visualized with yellow/orange colour (arrows). A, B, C, D x400. E Zoom.

**Figure 12:**
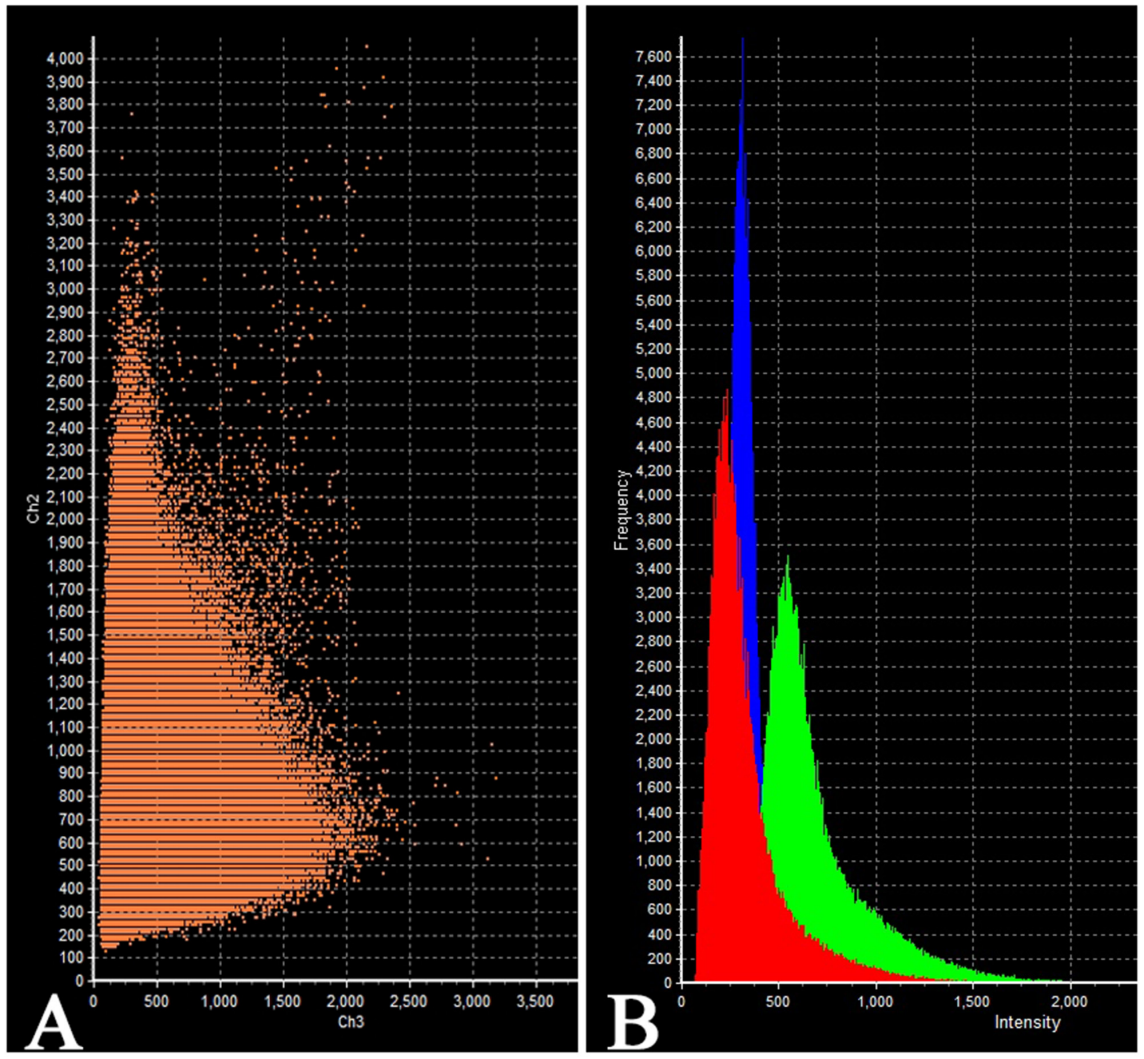
Laser Confocal Scanning Microscopy of the normal anterior pituitary **(A)** Scatterplot of red (Adrenocorticotropic hormone) and green (Prolactin) pixel intensities of pituitary cells. **(B)** Intensity Histogram of red (Adrenocorticotropic hormone), green (Prolactin) and blue (DAPI) fluorescence.

The morphometric study based on light microscopy showed the co-expression of prolactin and ACTH in 9,2±9,2% of the cells on the average (between 2 and 25%, Figure 10). LCSM revealed a prominent hormone co-expression in 34% of the cells of the pars distalis (Figure 11, 12).

**Figure 13:**
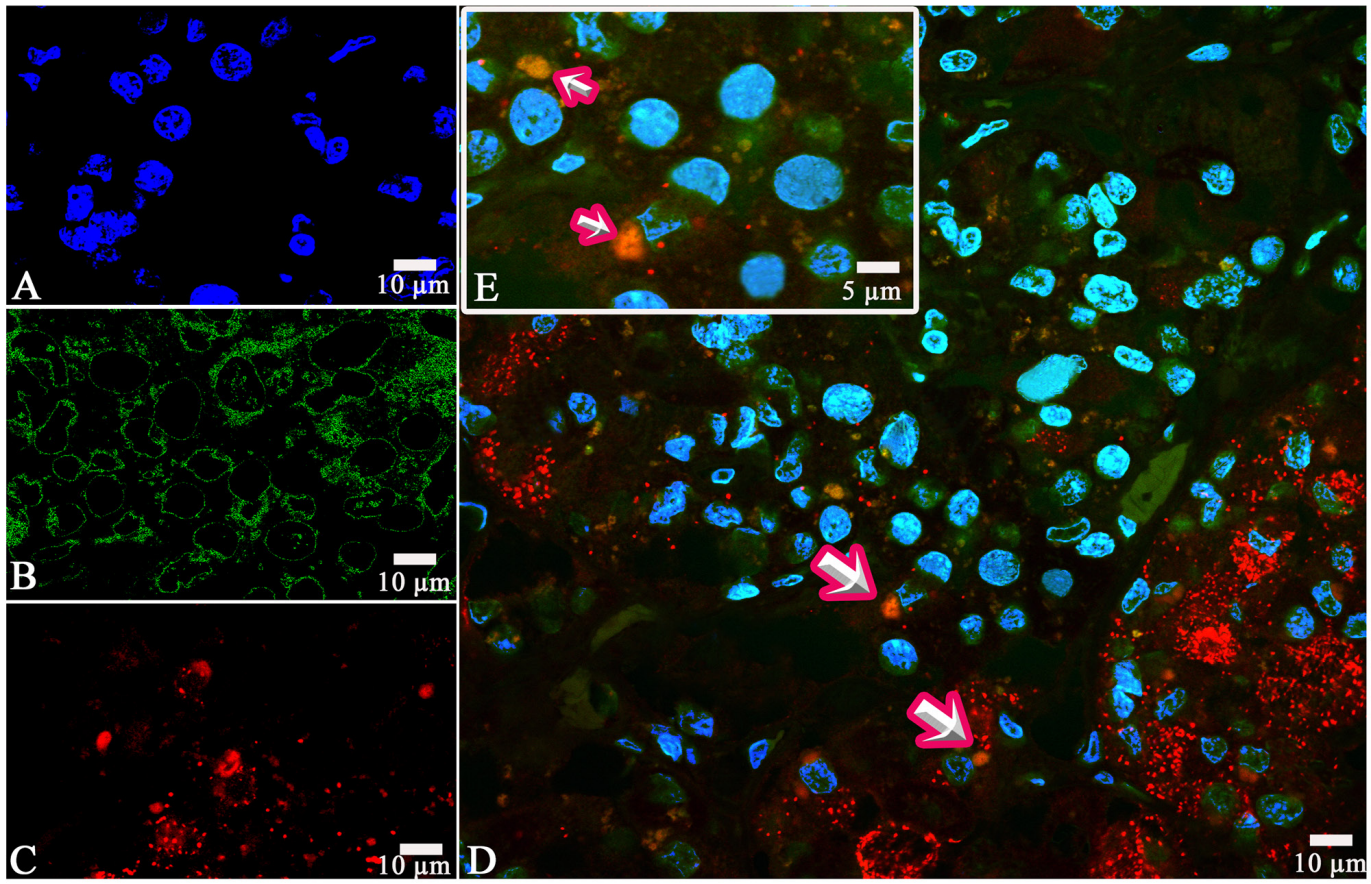
Laser Confocal Scanning Microscopy of the normal anterior pituitary **(A)** Blue fluorescence of cell nuclei (DAPI). **(B)** Green fluorescence of Growth hormone. **(C)** Red fluorescence of Adrenocorticotropic hormone. **(D, E)** Hormone co-expression is visualized with yellow/orange colour (arrows). A, B, C, D x400. E Zoom.

**Figure 14:**
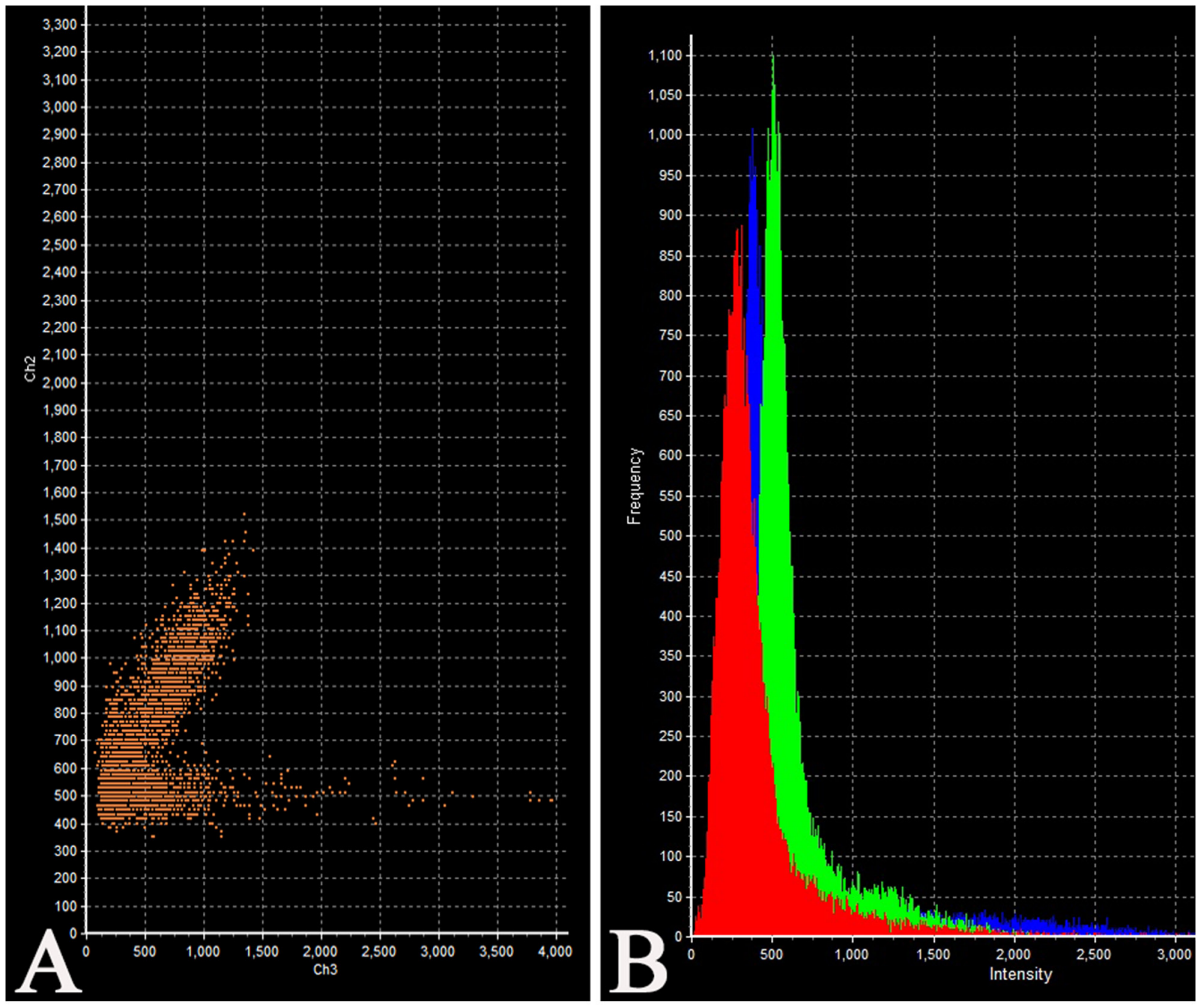
Laser Confocal Scanning Microscopy of the normal anterior pituitary **(A)** Scatterplot of red (Adrenocorticotropic hormone) and green (Growth hormone) pixel intensities of pituitary cells. **(B)** Intensity Histogram of red (Adrenocorticotropic hormone), green (Growth hormone) and blue (DAPI) fluorescence.

**Figure 15:**
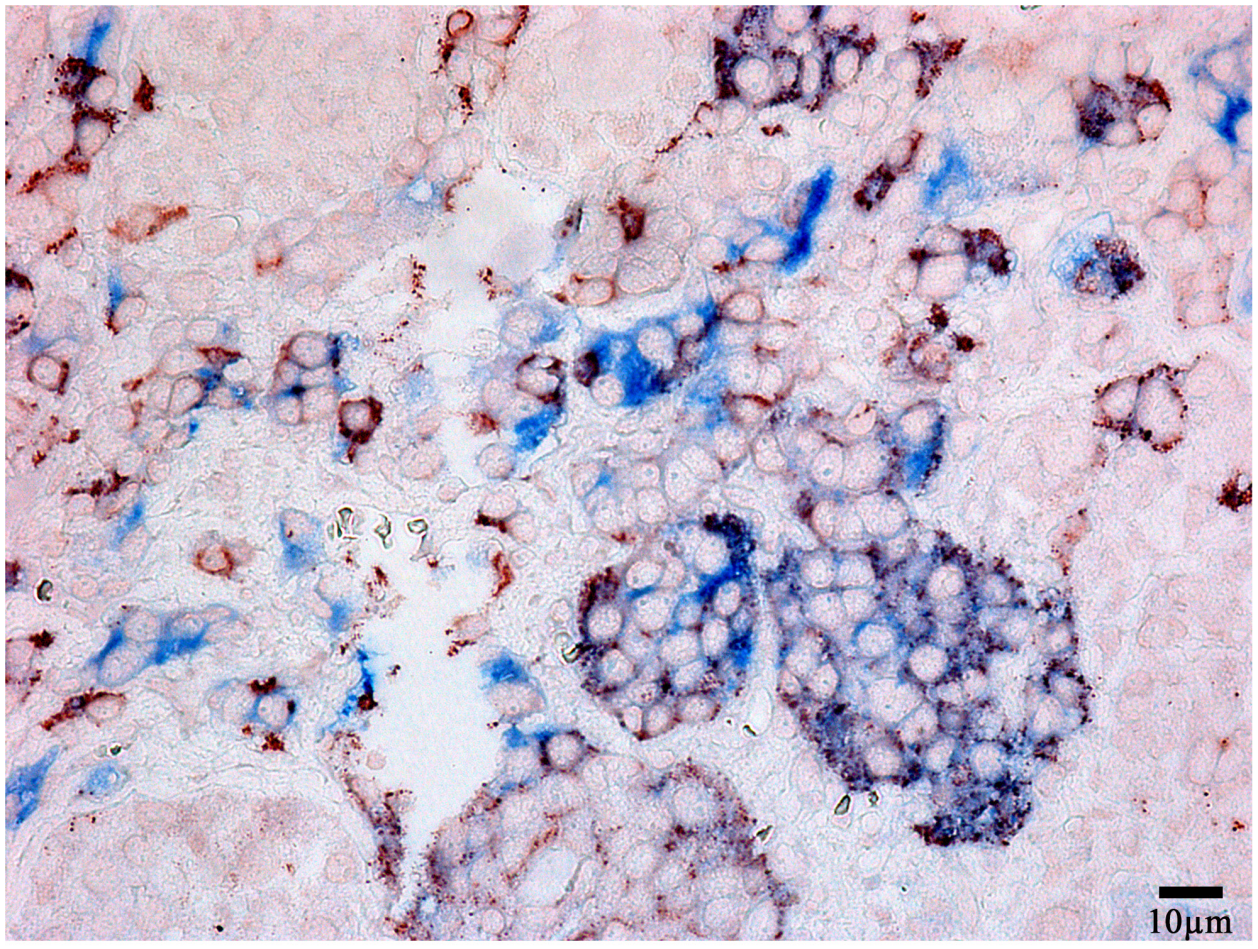
Double-staining immunohistochemistry of the normal anterior pituitary Growth hormone/Luteinizing hormone, x 400. Growth hormone is visualized with red colour, LH with blue colour, hormone co-expression with marron.

**Figure 16:**
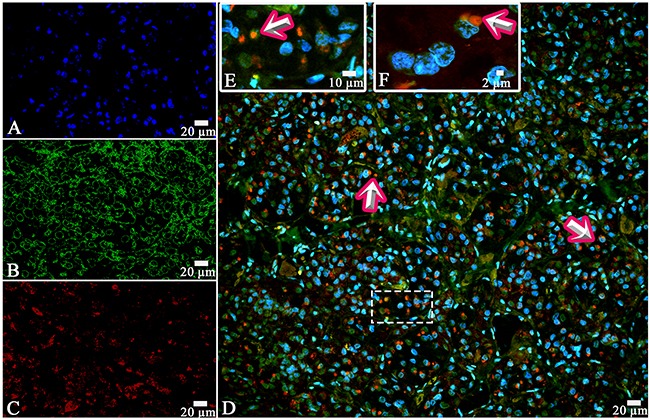
Laser Confocal Scanning Microscopy of the normal anterior pituitary **(A)** Blue fluorescence of cell nuclei (DAPI). **(B)** Green fluorescence of Growth hormone. **(C)** Red fluorescence of Follicle-stimulating hormone. **(D, E, F)** Hormone co-expression is visualized with yellow/orange colour (arrows). A, B, C, D x400. E Zoom. F x600.

**Figure 17:**
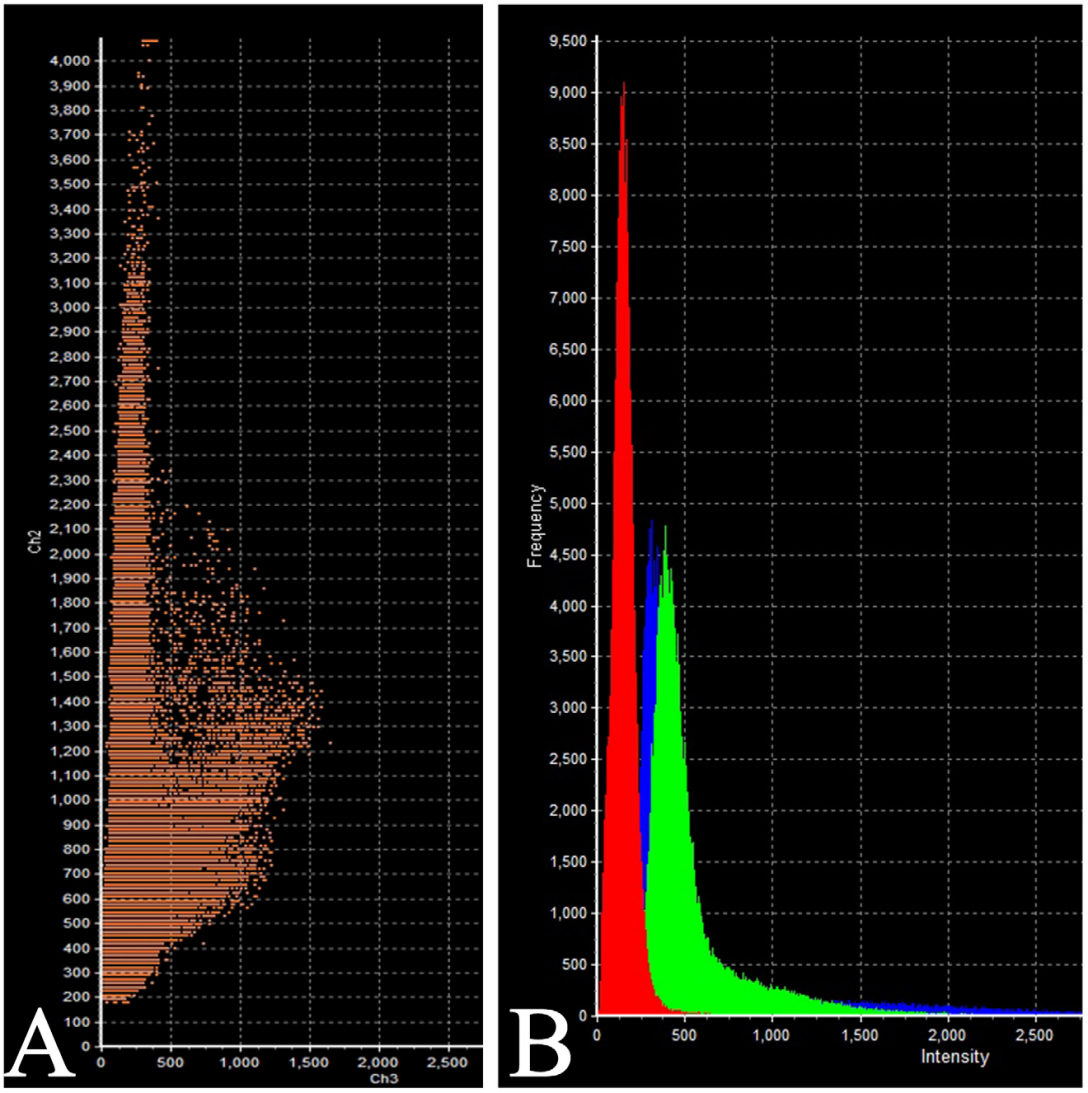
Laser Confocal Scanning Microscopy of the normal anterior pituitary **(A)** Scatterplot of red (Follicle-stimulating hormone) and green (Growth hormone) pixel intensities of pituitary cells. **(B)** Intensity Histogram of red (Follicle-stimulating hormone), green (Growth hormone) and blue (DAPI) fluorescence.

The co-expression of GH and ACTH was observed in the same cells of anterior pituitary in all the cases. The co-expression coefficient varied from 0,2 to 17%. Hormone co-expression was observed in 7,3±6,3% of the cells under a light microscope. LSCM displayed a prominent co-expression of GH/ACTH in 55% of the cells (Figure 13, 14). The co-expression coefficient of GH and LH varied from 0.4 to 24% and was 11,7±8,4% on the average (Figure 15). Co-expression of these hormones was observed in the same cells of the anterior pituitary in all the cases. Co-expression of GH and TSH was also observed in all the cases. The average co-expression coefficient was 6,4±5,4%, it varied from 0.2 to 15%. Co-expression of GH and FSH in the same cells of the anterior pituitary was determined in 8 of 10 cases (Figure 16, 17). Double-staining was not found in 2 patients: the 52-year-old man died of pneumonia and the 53-year-old woman diagnosed with stomach cancer died of pulmonary embolism. In the other 8 cases the co-expression coefficient varied between 8 and 25%, on the average it was 12,8±10,8%. The most prominent expression was observed in 40% of the cells per field under CLSM.

TSH and FSH were co-expressed in the same cells of anterior pituitary in all the 10 cases. The average co-expression coefficient was 1,0±0,8%, it varied between 0,1 and 4,3%. The cells co-expressed hormones were diffusely dispersed all over the anterior pituitary. TSH and ACTH were co-expressed in 8 of the 10 cases. Co-expression TSH and ACTH was not observed in the same above-mentioned 2 patients (the 52-year-old man died from pneumonia and the 53-year-old woman diagnosed with stomach cancer died because of pulmonary embolism). In 8 cases the average co-expression coefficient was 1,1±1,4%, it varied between 0,1 and 3%. The co-expression of TSH and LH was also observed in 8 of10 cases, in 0,86±1,04% of the cells on the average (from 0,2 to 2%).

**Figure 18:**
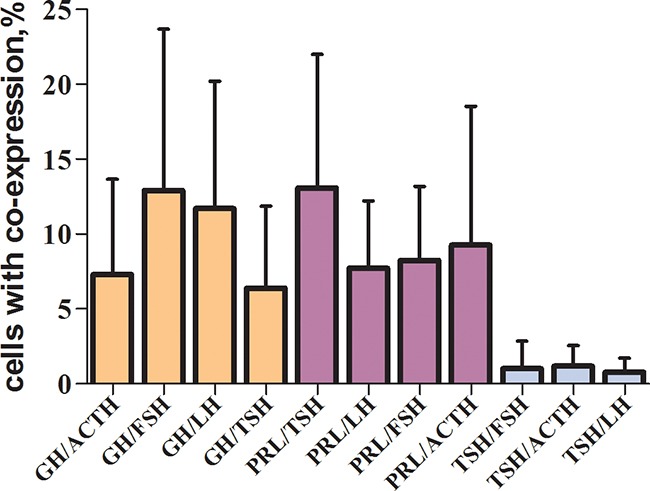
Average co-expression coefficients of hormones of the normal anterior pituitary as detected by light microscopy (double-staining immunohistochemistry) GH - growth hormone, ACTH - adrenocorticotropic hormone, FSH - follicle-stimulating hormone, TSH - thyroid-stimulating hormone, LH - luteinizing hormone.

**Figure 19:**
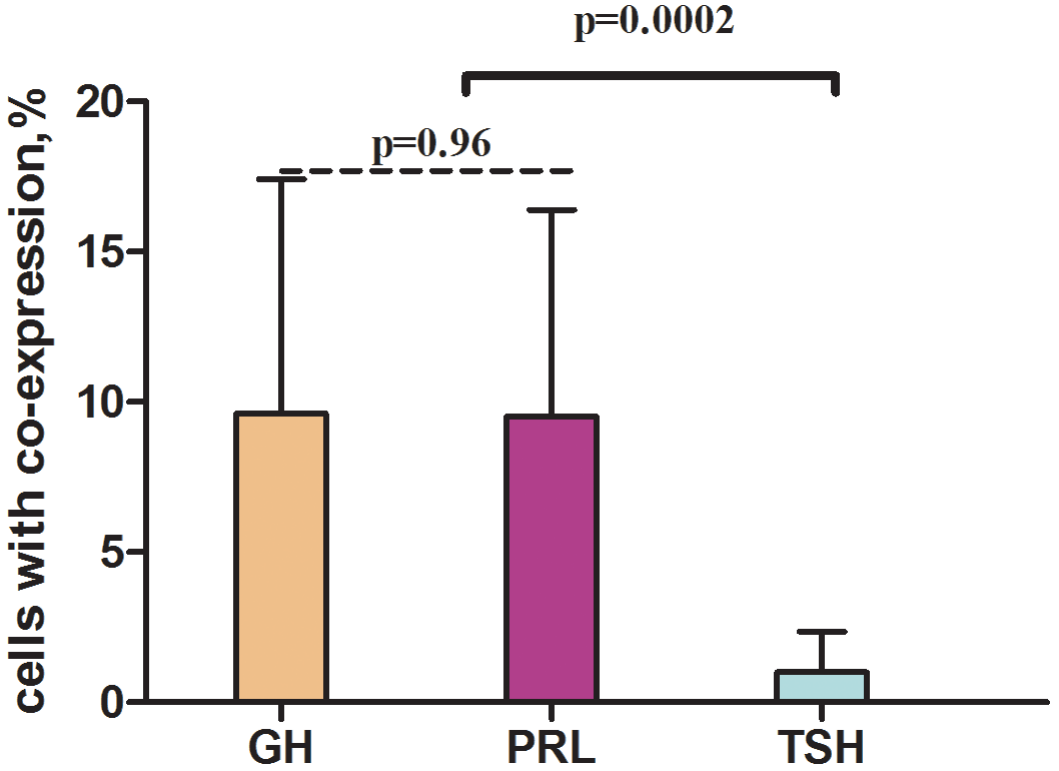
Average co-expression coefficients of GH - growth hormone, TSH - thyroid-stimulating hormone, PRL - prolactin

Having compared the average co-expression coefficients of prolactin, GH and TSH with other hormones, we found out that the co-expression coefficient of TSH and the other hormones was significantly the least (9,5±6,9%; 9,6±7,8%; 1,0±1,3% correspondingly, Figures 18, 19).

To sum up, double-staining immunohistochemistry and CLSM have shown that prolactin and each of the other hormones were co-expressed in all 10 cases. In the same two cases we have not observed coexpression of GH with FSH and TSH with LH and ACTH respectively.

## DISCUSSION

Using double-staining immunohistochemistry and CLSM for the first time we showed that the normal pituitary gland cells in adult humans can co-express prolactin, GH and TSH with other hormones, which refutes the concept “one cell type – one hormone”, as had been accepted before [6, 7].

Electron microscopic immunocytochemistry and laser scanning confocal microscopy revealed co-localization GH and TSH in the same cells in the anterior pituitary in adult rats with methimazole-induced hypothyroidism [8]. Co-localization of GH and α subunit together with gonadotropin β subunits was observed in some cells in the normal adult anterior pituitary gland [9]. Although the presence of more than one hormone is difficult to explain, advanced research methods and techniques have shown that precursor cells can differentiate into cells of various types. Corticotrophs are assumed to arise as a lineage distinct from that of the other pituitary cells [10, 11]. It should be stressed that plurihormonal adenomas often secrete GH, prolactin and ACTH simultaneously or a combination of one of these hormones and TSH. ACTH-secreting tumours can be also combined with gonadotrophin-secreting ones [12, 13, 14]. In all the cases under study the cells with the co-expression of ACTH and prolactin, GH or TSH were diffusively scattered in the anterior pituitary. Laser scanning confocal microscopy showed that the most prominent co-expression of ACTH/prolactin and ACTH/GH made up 34 and 55% correspondingly in some high-power fields. It is considered to be likely that somatotrophs, lactotrophs, thyrotrophs and gonadotrophs share common transcription factors. That is especially true for somatotrophs and lactotrophs, because, in contrast to other cell types which function independently, lactotrophs have a strong dependence on somatotrophs/lactotrophs and somatotrophs are often affected together [15].

Several different transcription factors regulating the transformation of pituicyte precursors into mature secretory cells have been identified. They are T-pit, RPx/Hesx-1, Ptx1, Ptx2, Lhx3/P-lim, Prop-1, Pit-1, SF-1, NeuroD-1, GATA-2 [16, 17]. For example, the GATA-2 is responsible for differentiation of gonadotropes and thyrotropes. NeuroD1 regulates POMC gene expression in corticotropes. Pit-1-dependent lineage includes stem somatotrophs, thyrotrophs, mammosomatotrophs, somatotrophs and prolactotrophs. In view of the fact that the pool of adenohypophyseal cells constantly replenishes, not only terminally differentiated but intermediate cytogenetic forms as well are constantly present in the anterior pituitary. These intermediate cytogenetic forms are likely to co-express several hormones simultaneously.

Along with transcription factors, cofactors (such as membrane receptors for hypothalamic hormones and estrogen nuclear receptors), growth factors, cell migration regulation factors, proliferation factors and differentiation factors are involved in the development of normal adenohypophysis and pituitary adenomas [18, 19, 20]. The growth and functions of adenohypophysis are regulated by hypothalamic hormones, peripheral hormones and intrapituitary growth factors and cytokines [21]. Multiple studies prove the fact that the regulation of hormones secretion in adenohypophysis is a complicated process influenced by numerous factors. It might be the reason we did not observe the co-expression of TSH and GH, TSH and ACTH, GH and FSH in oncology patients.

Considerable information on the pituitary adenoma aetiology has been derived from transgenic animal models, which may not accurately and universally reflect human pathophysiology and tumour pathogenesis [22]. Our study differs in the fact that original plurihormonality in the normal anterior pituitary cells has been demonstrated in the adult human.

In our opinion, the constant presence of a certain percentage of plurihormonal cells in the normal anterior pituitary throughout life, in certain abnormal circumstances (as affected by different pathological factors), makes the most obligate the development of plurihormonal adenomas and not monohormonal ones. It is much more logical to assume that suppression of transcription factors results in monohormonal adenomas.

Our study has given conclusive evidence that plurihormonality of normal anterior pituitary is an actually existing phenomenon. Identification of different hormones along with transcription factors in pituitary adenomas will enable to find new ways to improve both diagnostic process and targeted treatment of neuroendocrine tumours.

## MATERIALS AND METHODS

We studied 10 pituitary glands of 4 females and 6 males with cardiovascular and oncological diseases. The patients aged 48-63 years (the average age was 56,5±4,8 years, the clinical profile of patients is represented in Table 3) died from acute heart failure, pulmonary embolism, pneumonia or cancer progression. In all the cases the pituitary gland was removed within 2-12 hours after the death, its sizes were measured and it was fixed in 10% neutral buffered formalin and then embedded in paraffin. The paraffin sections of all 10 specimens were stained with hematoxylin and eosin. The Gordon and Sweet's silver staining method was used for reticular fibers. Immunohistochemistry using antibodies against ACTH, GH, TSH, prolactin, FSH and LH was carried out in all 10 specimens and scanned with Aperio Digital Pathology Scanner (Leica, Germany) and then the immunohistochemistry stained tissue sections were compared with different antigens. Double staining immunohistochemistry using 11 hormone combinations was performed in all the cases. These combinations were: prolactin/TSH, prolactin/LH, prolactin/FSH, prolactin/ACTH, GH/TSH, GH/LH, GH/FSH, GH/ACTH, TSH/LH, TSH/FSH, TSH/ACTH. Confocal Laser Scanning Microscopy (Olympus FV1000D, Japan) with a mixture of primary antibodies, namely ACTH/prolactin, FSH/prolactin, TSH/prolactin, ACTH/GH, and FSH/GH, was performed in 2 cases (two first patients in Table 3).

**Table 3:** Clinical characteristics of the patients

№	Sex	Age	Disease	Cause of death	Pituitarymeasurements, length × width ×height (mm)
1	Male	55	Ischaemic heart disease	Heart failure	14×10×6
2	Female	59	Ischaemic heart disease	Heart failure	11×10×6
3	Female	53	Stomach cancer	Pulmonary embolism	11×7×7
4	Female	63	Ischaemic heart disease	Pulmonary embolism	9×5×5
5	Male	52	Leukemia	Pneumonia	15×6×6
6	Female	60	Rheumatic heart disease	Heart failure	12×6×7
7	Male	64	Ischaemic heart disease	Heart failure	9×8×6
8	Male	54	Dilated cardiomyopathy	Pulmonary embolism	10×6×7
9	Male	57	Ischaemic heart disease	Heart failure	9×7×6
10	Female	48	Uterine cancer	Cancer intoxication	11×8×8

### Immunohistochemical study

**Figure 20:**
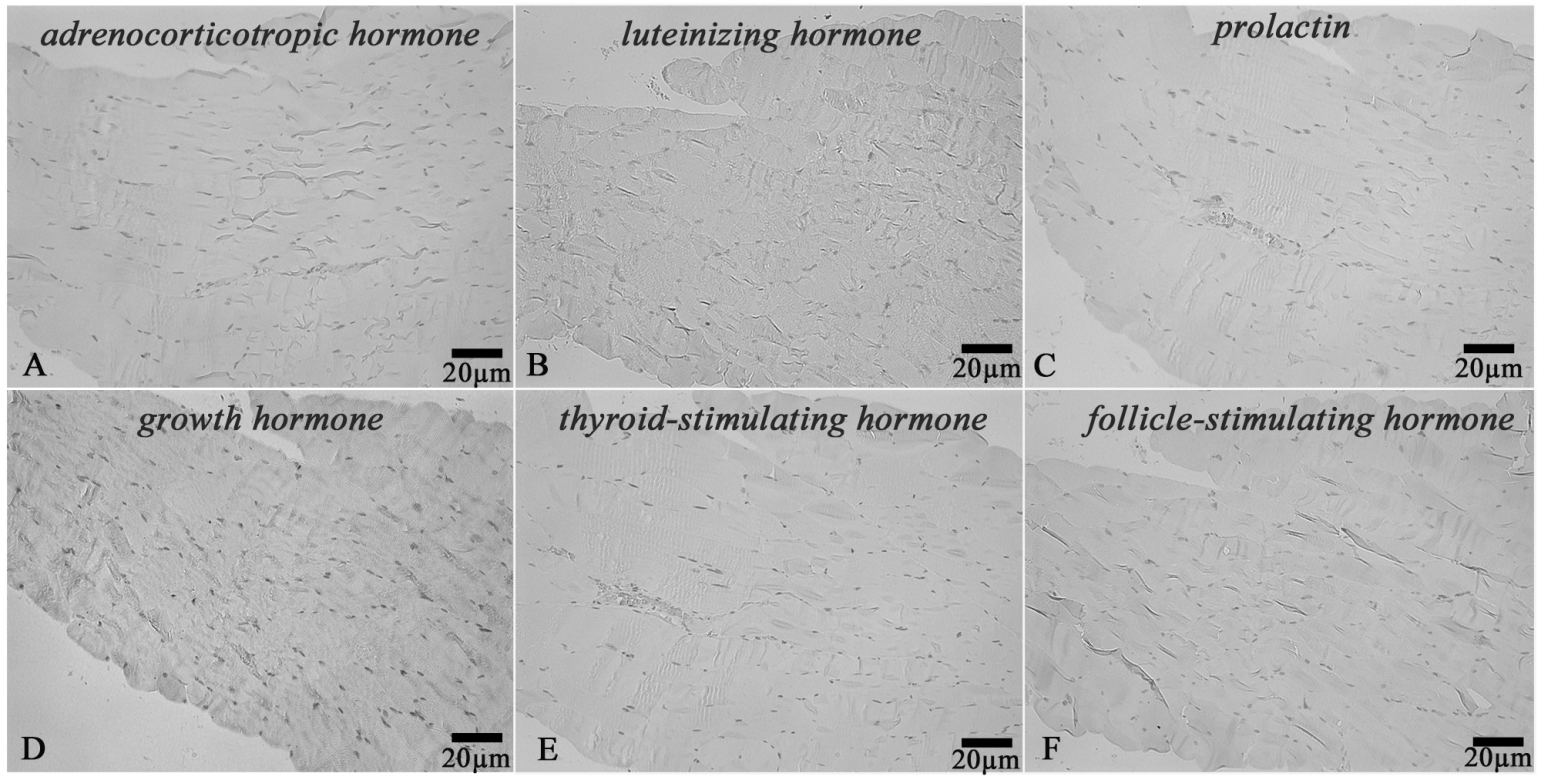
Skeletal muscle Negative control for immunohistochemical staining with primary antibodies to: **(A)** Adrenocorticotropic hormone, **(B)** Luteinizing hormone, **(C)** Prolactin, **(D)** Growth hormone, **(E)** Thyroid-stimulating hormone, **(F)** Follicle-stimulating hormone, x200.

Paraffin embedded sections were first deparaffinized with xylene and rehydrated in a graded ethanol series. To inactivate endogenous peroxidase the sections were treated with 3% hydrogen peroxide for 5 minutes at room temperature and then washed in distilled water. Antigen retrieval was performed with Tris-EDTA buffer (pH 9.0) at 95-98°C for 35 min (TRS 9.0, Dako, Denmark). Then the sections were cooled to room temperature. Subsequently they were washed twice in Tris-Buffered Saline with Tween 20 (TBST) each for 5 minutes (TBS, Dako, Denmark). Primary antibody incubation was carried out in a container with wet filter paper at room temperature for 30 minutes. After that the sections were washed twice in TBST. The specimens were incubated with EnVision Detection Systems Rabbit/Mouse kit (Dako, Denmark) at room temperature for 30 min. Then the sections were washed twice in TBST, each for 5 minutes. The coloured DAB reaction products were visualized directly by light microscopy. After washing in distilled water, the sections were counterstained with hematoxylin for 2 minutes, dehydrated, and then mounted using a permanent mounting medium (Polystyrol, BioMount, Italy). Skeletal muscle was used as a negative control for each immunohistochemical test (Figure 20).

For immunohistochemical staining we used primary antibodies to:

- mouse monoclonal ACTH antibody at a dilution range of 1:500 (clone AH26, Diagnostic BioSystems, Netherlands);
- rabbit polyclonal TSH antibody (RTU, Cell Marque, USA);
- mouse monoclonal FSH antibody at a dilution range of 1:100 (clone C10, DAKO, Denmark);
- mouse monoclonal LH antibody at a dilution range of 1:500 (clone C93, DAKO, Denmark);
- rabbit polyclonal GH antibody at a dilution range of 1: 100 (BioGenex, USA);
- rabbit polyclonal prolactin antibody at a dilution range of 1: 700 (DAKO, Denmark).

For double immunohistochemical stain the deparaffinized and dehydrated pituitary sections were treated with TRIS EDTA (pH 9.0) at 95-98C° in a water bath for 25 minutes and cooled down at room temperature for 20 minutes and then washed in distilled water. Next the specimens were incubated in Hydrogen Peroxidase Block solution at room temperature for 10 minutes. After that the sections were washed twice in TBST, each for 5 minutes.

To reduce nonspecific background staining the tissue specimens were incubated with UltraVBlock for 10 minutes at room temperature. Each cocktail of primary antibodies was incubated with MultiVision anti-rabbit/HRP + anti-mouse/AP polymer cocktail (Thermo Scientific, UK) at room temperature for 30 minutes and washed twice in TBST. LVBlue and LVRed working solutions were applied to incubate the sections for 10 minutes each. Then one part of anterior pituitary hormone antigens resulted in blue, another part gave red colour (Table 4) and the double-stained cells (their co-expression) were coloured in maroon. The co-expression coefficient was defined as the ratio of the double-stained cells to the single-stained ones in percent per 10 high power fields at 400x magnification. Co-expression coefficients of two antibodies were determined using image analysis software Image Scope Color M (Russia).

**Table 4:** Hormone co-expression in the same anterior pituitary cells

Hormones	PRL	GH	ACTH	LH	FSH	TSH
PRL	++++		+++/−	+++/−	+++/−	+++/−
GH		++++	+++/−	--/++	+++/−	---/+
ACTH	+++/−	+++/−	++++			---/+
LH	+++/−	--/++		++++		++/−-
FSH	+++/−	+++/−			++++	---/+
TSH	+++/−	--/++	---/+	---/+	---/+	++++

*blank cells – no information available on double-staining

PRL - prolactin, GH - growth hormone, ACTH - adrenocorticotropic hormone, LH - luteinizing hormone, FSH - follicle-stimulating hormone, TSH - thyroid-stimulating hormone

### Confocal laser scanning microscopy

To determine the ratio of required markers we used the following mixtures of primary antibodies for each specimen: ACTH/prolactin, FSH/prolactin, TSH/prolactin, ACTH/GH, TSH/GH. The deparaffinized and dehydrated pituitary sections were 4 to 10 μm thick. Heat-induced epitope retrieval (HIER) with 10 mM citrate buffer (pH 6,0) was performed using a pressure cooker. PBS buffer and Tween 20 were used as a wash buffer. Then the sections were incubated for 30 minutes with the Blocking Serum at room temperature. After washing, the first primary TSH antibodies (clone M1A10, Abcam, at a dilution range of 1:300), FSH antibodies (clone C10, DAKO, at a dilution range of 1:100) and ACTH antibodies (clone AH26, Diagnostic BioSystem, at a dilution range of1:300) were applied and then these sections were incubated for 1 hour at room temperature. We used Alexa Fluor 647® secondary antibodies (Abcam, UK). After additional washing, the sections were incubated with the second primary prolactin antibodies (rabbit polyclonal antibody, DAKO, at a dilution range of1:700) or GH antibodies (rabbit polyclonal antibody, BioGenex, at a dilution range of 1:100) for 1 hour at room temperature. We used Alexa Fluor 488® secondary antibodies (Abcam, UK). After washing, the sections were counterstained with DAPI (appliChem). Dako Mounting Medium was used for mounting all the tissue specimens. As a result the first antibodies showed up in red fluorescence; the second antibodies gave green fluorescence; the double stained resulted in yellow-orange fluorescence and the contrasted nuclei in blue fluorescence. The Olympus FV1000D confocal laser scanning microscope was used for fluorescent observation of specimens. We evaluated the intensity and colocalization of hormone expression (fluorescence). Micrographs of 2 anterior pituitary glands (per 5 high power fields at 400x magnification) displaying most prominent double staining were taken for all 5 hormone combinations. The co-expression coefficient of hormones was defined as the ratio of the double-stained cells to the single-stained ones, expressed in percentage. The co-expression coefficients of hormones were determined using image analysis software (Image Scope Color M, Russia).

### Statistical analysis

All statistical analysis was performed using Statistica software (V10.0, StatSoft, USA). All continuous variables are expressed as mean ± SD and categorical variables as number of subjects (%). Continuous variables were compared using analysis of variance and Student's t-test as appropriate. Categorical variables were compared using Fisher's exact test. Significant differences between groups were considered to be those at p<0,05.

## Abbreviations

TSH: thyroid-stimulating hormone
LH: luteinizing hormone
FSH: follicle-stimulating hormone
ACTH: adrenocorticotropic hormone
GH: growth hormone
LCSM: laser confocal scanning microscopy
POMC: pro-opiomelanocortin
